# Rosuvastatin Alleviates Intestinal Injury by Down-Regulating the CD40 Pathway in the Intestines of Rats Following Traumatic Brain Injury

**DOI:** 10.3389/fneur.2020.00816

**Published:** 2020-08-11

**Authors:** Yangchun Hu, Xiaojian Wang, Lei Ye, Chao Li, Weiwei Chen, Hongwei Cheng

**Affiliations:** Department of Neurosurgery, First Affiliated Hospital of Anhui Medical University, Hefei, China

**Keywords:** rosuvastatin, CD40, NF-κB, intestinal injury, traumatic brain injury

## Abstract

Statins have been reported to suppress CD40 expression and nuclear factor (NF)-κB activation, which are both up-regulated in the intestines following traumatic brain injury (TBI)-induced intestinal injury. In this study, we aimed to investigate the effects of the statin rosuvastatin on post-TBI jejunal injury in rats, focusing on potential mechanisms involving the CD40/NF-κB signaling pathway. The jejunal CD40 expression was determined by western blotting. The DNA-binding activity of NF-κB was assessed by electrophoretic mobility shift assays (EMSAs). The tumor necrosis factor (TNF)-α and interleukin (IL)-1β levels were assessed by enzyme-linked immunosorbent assays (ELISAs). The severity of the jejunal mucosal injury was assessed by hematoxylin and eosin (HE) staining and histopathological evaluation. We found that the post-TBI upregulation of both CD40 expression and NF-κB activity in the jejunal tissues were significantly inhibited by rosuvastatin, while the post-TBI expression of TNF-α and IL-1β was significantly suppressed by rosuvastatin. In addition, rosuvastatin significantly ameliorated TBI-induced effects on the villus height, crypt depth, and villous surface area. Rosuvastatin suppressed TBI-induced intestinal injury in rats, which may be associated with the blockade of the CD40/NF-κB pathway.

## Introduction

Traumatic brain injury (TBI) is a serious medical problem worldwide, with extremely high disability and mortality rates (1). Although intensive investigations of TBI have been carried out, researchers have focused mainly on the pathophysiologic processes of the brain injury itself. However, it is also important to realize that extracranial complications following the initial brain injury might, to some extent, impede treatment efficacy, and recovery. Therefore, understanding the etiology and underlying mechanisms of post-TBI extracranial complications is important (2).

Organ dysfunction, especially gastrointestinal dysfunction, has frequently been observed in TBI patients (3). In previous research, we demonstrated that TBI can induce marked damage to intestinal mucosal structures and barrier functions (4). Additionally, we found that TBI up-regulated the intestinal expression of CD40, nuclear factor (NF)-κB, and pro-inflammatory cytokines, which may play pivotal roles in the pathogenesis of acute intestinal mucosal injury (5–7). Several studies have suggested that the inflammatory responses mediated by NF-κB and other inflammatory cytokines are key factors in intestinal mucosal damage (8–10). Notably, both *in vivo* and *in vitro* studies have reported that statins regulate CD40 expression (11), block NF-κB activation (12, 13), and further exhibit anti-inflammatory properties, in addition to exhibiting lipid-lowering effects (14).

The statin rosuvastatin, a new 3-hydroxy-3-methylglutaryl coenzyme A (HMG-CoA) reductase inhibitor, has increased affinity for the active site of HMG-CoA reductase compared to other statins. The protective role of rosuvastatin in preventing ischemic injury has been clearly documented (15, 16). Yuji et al. reported that rosuvastatin reduced intestinal ischemia-reperfusion injury in animal models (17). This indicates that rosuvastatin is a potential candidate drug for protecting against post-TBI intestinal injury. Hence, in this study, we aimed to investigate whether rosuvastatin treatment could regulate the CD40/NF-κB signaling pathway, and tried to clarify the potential role of this pathway in post-TBI intestinal injury.

## Materials and Methods

### Rat Model of TBI

Male Sprague-Dawley rats, weighing about 250–300 g, were purchased from the Experimental Animal Center of Anhui Medical University. They were housed at about 25°C in a controlled environment with 12 h of artificial light per day. They were randomized into three groups: the sham operation + normal saline group (SN, *n* = 6), TBI + normal saline group (TN, *n* = 6), and TBI + rosuvastatin group (TR, *n* = 6).

TBI was induced using a modified version of Feeney's weight-drop model technique (18). Briefly, the rats were anesthetized with 4% isoflurane and the anesthetic effect was maintained with 2% isoflurane (0.6 L/min) delivered by a small-animal anesthetic machine. Thereafter, a right parietal craniotomy (5 mm in diameter) was performed at 1 mm posterior and 2 mm lateral to the bregma. A steel rod (weighing 40 g with a flat end and a diameter of 4 mm) freely fell from a height of 25 cm onto the exposed intact cranial dura to produce a standardized parietal contusion. The rod was allowed to compress the tissue a maximum of 5 mm. The rats in the sham operation group were anesthetized, mounted in the stereotaxic apparatus and had their scalps cut and sutured but did not undergo trephination.

At 30 min after the TBI, rosuvastatin (AstraZeneca UK, Ltd., London, UK) was dissolved in isotonic normal saline and administered at 30 mg/kg intraperitoneally. In the SN group, an equivalent amount of saline was administered intraperitoneally under the same time condition.

All applicable international, national, and/or institutional guidelines for the care and use of animals were followed.

### Preparation of Jejunal Tissues

The rats were decapitated 24 h after the TBI to conduct tissue assays. A-3 cm segment of the mid-jejunum was obtained, flushed with ice-cold saline, and opened longitudinally. For the histopathological evaluation, jejunal tissues were immersed in 4% buffered formalin.

### Western Blotting (WB) Analysis

The total proteins of the jejunal tissue homogenates were extracted using a radioimmunoprecipitation assay (RIPA) buffer kit (Beyotime Biotechnology, Shanghai, China) according to the manufacturer's protocol. They were then measured using a bicinchoninic acid (BCA) protein quantification kit (Thermo Scientific, Waltham, MA, USA), according to the manufacturer's protocol. The CD40 protein levels were assessed as previously described (7). Briefly, the proteins were separated by 10% sodium dodecyl sulfate polyacrylamide gel electrophoresis and then transferred to polyvinylidene difluoride membranes (Millipore Corp., Bedford, MA, USA). The membranes were blocked with 5% non-fat milk (w/v) dissolved in Tris-buffered saline with Tween 20 for 1 h at room temperature. The proteins were then labeled with the following primary antibodies: anti-CD40 antibody (1:100; Santa Cruz Biotechnology, CA, USA) and anti-glyceraldehyde 3-phosphate dehydrogenase (GAPDH) antibody (1:1,000; Sigma, St Louis, MO, USA) at 4°C overnight. This was followed by incubation with secondary goat anti-mouse IgG (H+L) antibody (peroxidase/horseradish peroxidase conjugated; 1:1,000; E-AB-1001; Elabscience, Wuhan, China) for 1.5 h at room temperature. The optical density of the resulting bands was determined using UN-SCAN-IT graph digitizing software (UT, USA), with densitometry values normalized to the GAPDH values.

### Electrophoretic Mobility Shift Assay (EMSA)

We performed EMSA to detect the NF-κB DNA-binding activity as previously described (5). Briefly, a consensus oligonucleotide probe containing the DNA-binding site for NF-κB (5′-AGTTGAGGGGACTTTCCCAGGC-3′) was end-labeled with [γ-^32^P]-ATP (Free Biotech, Beijing, China) using T4-polynucleotide kinase. Competitive reactions were performed by adding a 100-fold excess of unlabeled NF-κB consensus oligonucleotide. HeLa nuclear extract was used as the positive control. Data were expressed as arbitrary densitometry units (ADU) obtained from the densitometric scans.

### Enzyme-Linked Immunosorbent Assay (ELISA)

The concentrations of tumor necrosis factor (TNF)-α and interleukin (IL)-1β in the jejunal tissue supernatants were determined using ELISA kits according to the manufacturer's protocols (TNF-α ELISA kit from Diaclone Research, Besançon, France; IL-1β ELISA kit from BioSource Europe SA, Nivelles, Belgium).

### Histopathological Evaluation

The formalin-fixed jejunal tissues were embedded in paraffin, sectioned at 4-μm thickness with a microtome, and stained with hematoxylin and eosin (HE). The villus height, diameter of the middle of the villus, and crypt depth in the tissues were determined using an HPIAS-1000 image analysis system (Champion Image Engineering Company, Wuhan, China). The villous surface area was calculated on the basis of the following formula: surface area = πdh (d, villus diameter; h, villus height). At least 10 well-oriented crypt-villus units per sample were assessed and average values were calculated by an independent pathologist who was blind to the animal grouping.

Additionally, pathology grades were determined based on the following criteria, as described in a previous study (19): 0: normal; 1: mild focal infiltration of the lamina propria; 2: mild infiltration of the lamina propria, multifocal, and mild glandular separation; 3: infiltration with multifocal mild edema; 4: mixed infiltration of the submucosa and lamina propria, extensive separation of glands, plaque enlargement, and edema.

### Statistical Analysis

We used SPSS (version 19.0. IBM Co., Ltd., Armonk, NY, USA) for the statistical analysis. Each parameter was expressed as mean ± SD and compared between groups using Kruskal-Wallis test or one-way analysis of variance (ANOVA), followed by Tukey's *post-hoc* test. *P* < 0.05 was considered statistically significant.

## Results

### CD40 Protein Levels in the Jejunal Tissues

**Figure 1:**
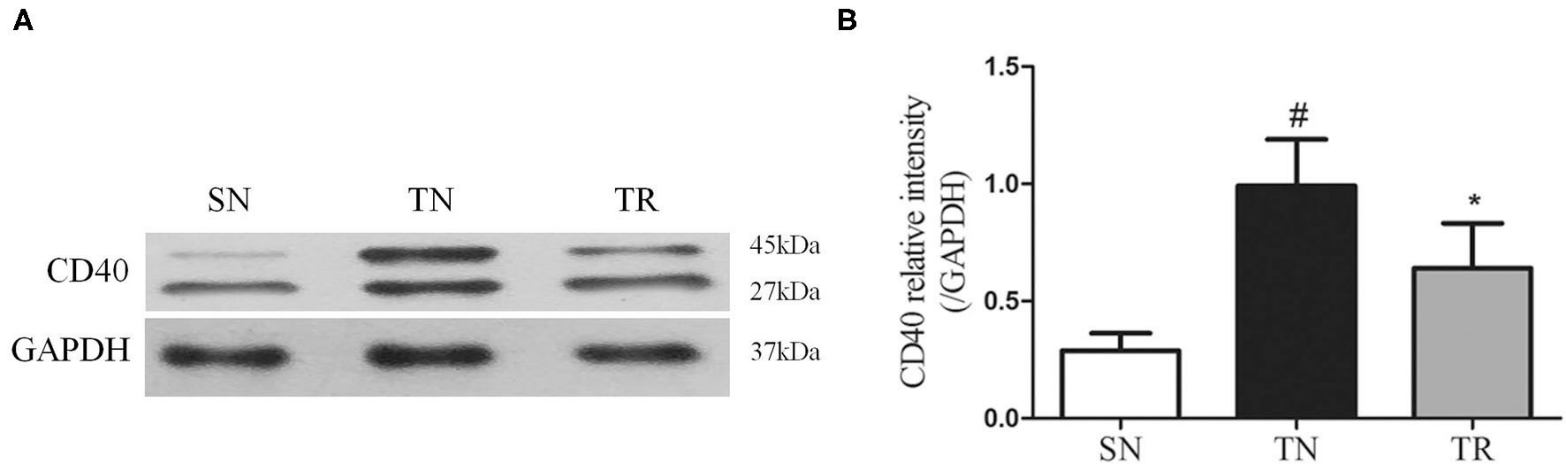
Western blotting analysis of CD40 protein expression. **(A)** Representative western blots of CD40 levels in the jejunal tissues in the SN, TN, and TR groups. **(B)** Quantitative analysis of the western blotting results for CD40. CD40 expression was up-regulated following traumatic brain injury (TBI; TN vs. SN), but the increased level of CD40 was suppressed by rosuvastatin (TR vs. TN). Bars represent mean ± SD (*n* = 6 per group). *P < 0.05 vs. SN group; ^#^*P* < 0.05 vs TR group. SN, sham operation + normal saline; TN, TBI + normal saline; TR, TBI + rosuvastatin.

CD40 protein levels were measured by WB. As shown in Figure 1, compared to the SN group, the level of CD40 protein significantly increased in the TN group (*P* < 0.01), while the rosuvastatin (in the TR group) significantly suppressed the CD40 protein level compared to that in the TN group (*P* < 0.05). The replicates of CD40 proteins in other 5 groups were provided as request (Supplementary Figure 1).

### NF-κB DNA-Binding Activity in the Jejunal Tissues

**Figure 2:**
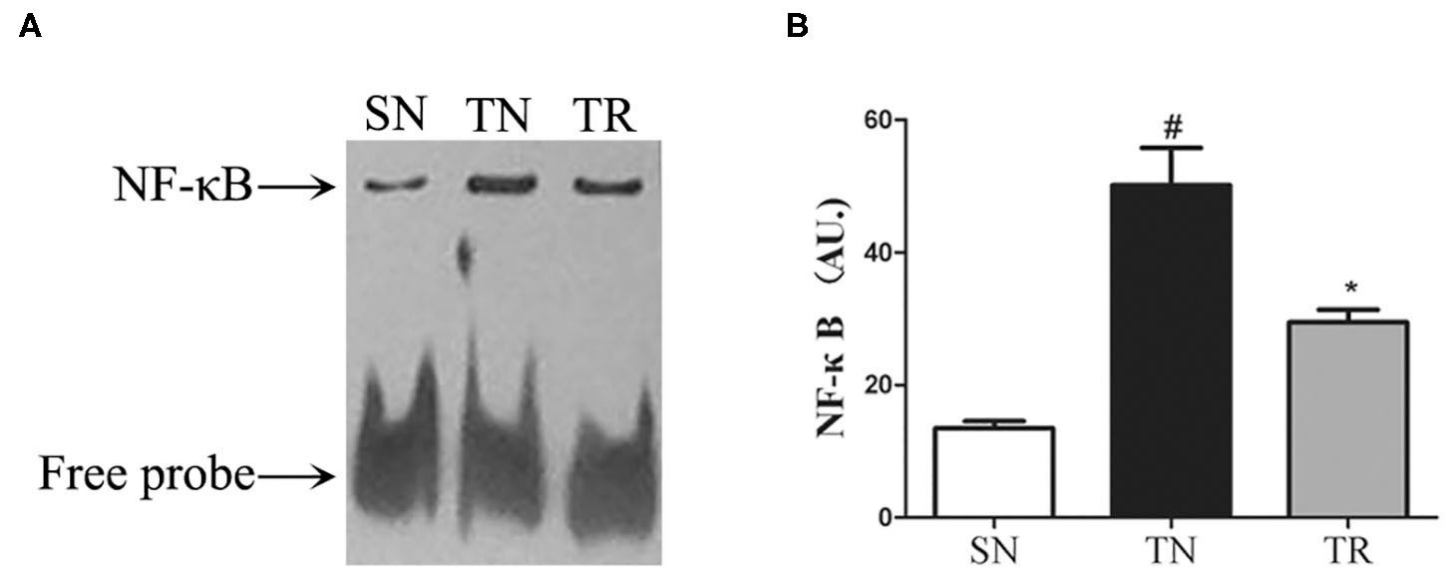
Electrophoretic mobility shift assays (EMSAs) of NF-κB DNA-binding activity. **(A)** EMSA results showing NF-κB DNA-binding activities in the SN, TN, and TR groups. **(B)** Quantitative analysis of the NF-κB DNA-binding activity. NF-κB DNA-binding activity increased following traumatic brain injury (TBI; TN vs. SN), but the increased activity was suppressed by rosuvastatin (TR vs. TN). Bars represent mean ± SD (*n* = 6 per group). *P < 0.05 vs. SN group; ^#^*P* < 0.05 vs TR group. SN, sham operation + normal saline; TN, TBI + normal saline; TR, TBI + rosuvastatin.

The results of EMSAs of NF-κB DNA-binding activity in the jejunal tissues are shown in Figure 2. Low NF-κB DNA-binding activity (i.e., weak EMSA autoradiography) was found in the SN group. In contrast, NF-κB DNA-binding activity was significantly up-regulated in the TN group compared to the SN group (*P* < 0.01), while it was significantly suppressed in the TR group compared to the TN group (*P* < 0.05).

### Concentrations of IL-1β and TNF-α in the Jejunal Tissues

**Figure 3:**
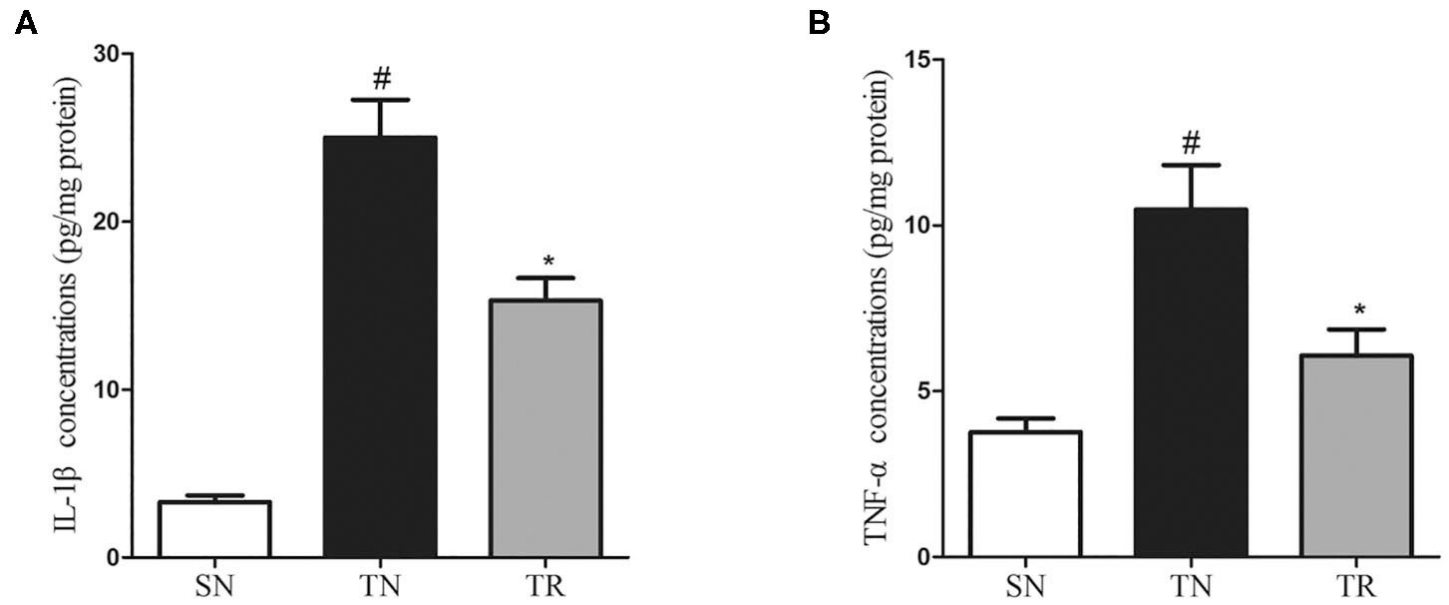
Changes in inflammatory mediators in jejunal tissues as determined by enzyme-linked immunosorbent assays (ELISAs). Traumatic brain injury (TBI) significantly increased the concentrations of IL-1β and TNF-α in rat jejunal tissues. In the TR group, the jejunal concentrations of IL-1β **(A)** and TNF-α **(B)** were markedly suppressed compared to those in the TN group. Bars represent mean ± SD (*n* = 6 per group). *P < 0.05 vs. SN group; ^#^*P* < 0.05 vs TR group. SN, sham operation + normal saline; TN, TBI + normal saline; TR, TBI + rosuvastatin.

ELISAs were performed to assess the concentrations of IL-1β and TNF-α in the jejunal tissues. The results showed that the concentrations of both IL-1β and TNF-α were extremely low in the SN group, and they were greatly enhanced in the TN group (*P* < 0.01). Rosuvastatin significantly suppressed the post-TBI concentrations of IL-1β and TNF-α in the jejunal tissues (*P* < 0.05) (Figure 3).

### Histopathological Evaluation

**Figure 4:**
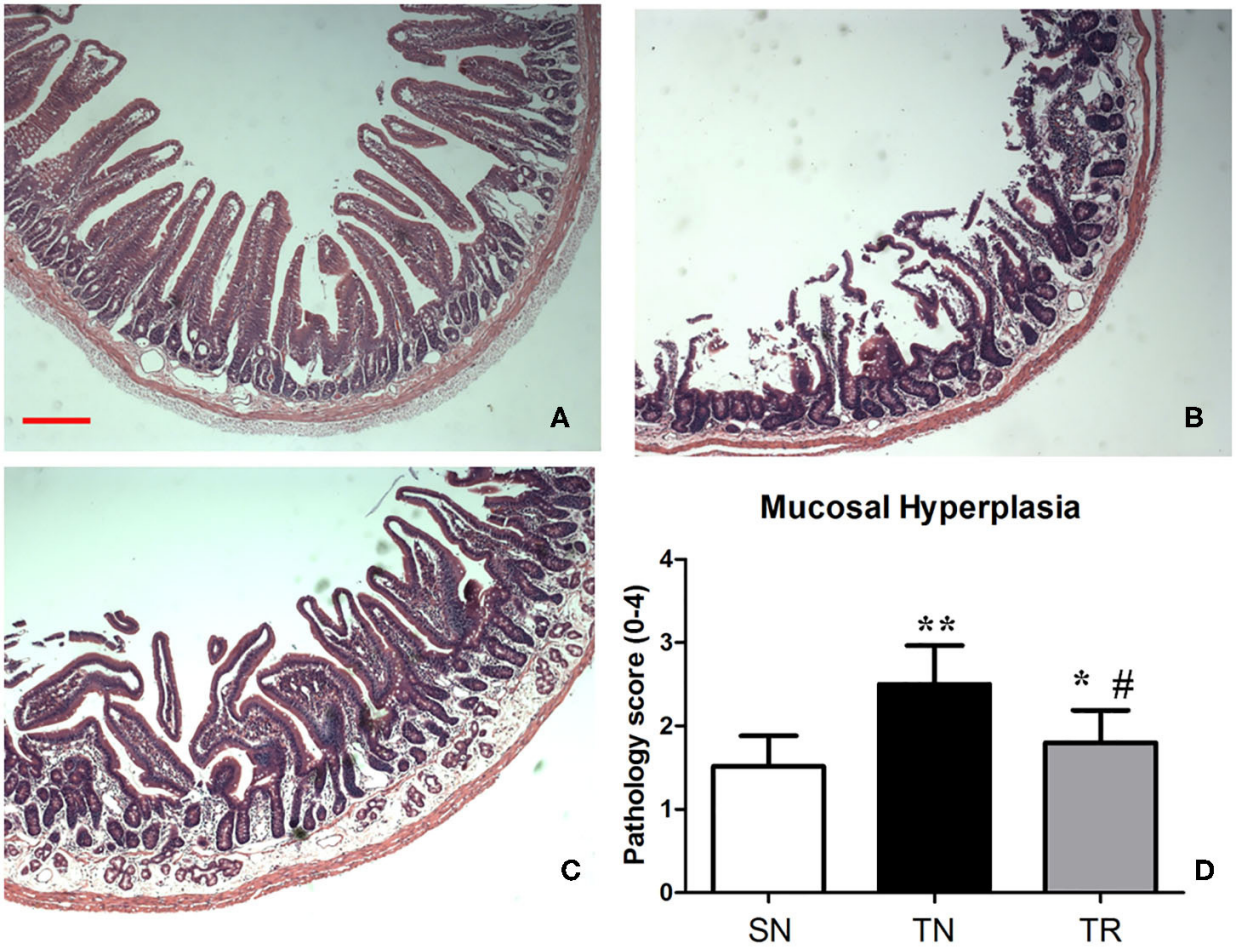
Hematoxylin and eosin (HE) staining of the mucosal structures of the jejunum. **(A)** Rats in the SN group exhibited normal mucosal architecture with intact villi. **(B)** Traumatic brain injury (TBI) resulted in shedding of epithelial cells, broken villi, focal ulcers, fusion between adjacent villi, dilation of central chyle duct, mucosal atrophy, and edema in the villus interstitium and lamina propria. **(C)** Rosuvastatin significantly suppressed the TBI-induced morphologic alterations of the jejunal mucosa. Scale bar = 200 μm. **(D)** Quantitative analysis of morphology of the jejunal mucosa with Kruskal Wallis tests. *P < 0.05, **P < 0.01 vs. SN group; ^#^*P* < 0.05 vs TN group. SN, sham operation + normal saline; TN, TBI + normal saline; TR, TBI + rosuvastatin.

Villus height, crypt depth, and villous surface area were determined as specific evaluation indices of mucosal damage. Histopathological assessment showed that the morphology of the jejunal mucosa was approximately normal in the SN group. TBI caused considerable damage to the mucosal structures. However, this damage was ameliorated by rosuvastatin administration (Figure 4). Quantitative analyses demonstrated that the villus height, crypt depth, and villous surface area significantly decreased in the TN group compared to the SN group (*P* < 0.01). In the TR group, these parameters were significantly increased compared to those in the TN group (*P* < 0.05) (Table 1).

**Table 1:** Changes in villous height, diameter, crypt depth, and surface area of mucosa.

**Groups**	**Villous height (μm)**	**Villous diameter (μm)**	**Crypt depth (μm)**	**Surface area (mm^**2**^)**
SN	241.4 ± 28.1	47.4 ± 7.5	81.6 ± 12.6	0.0362 ± 0.0041
TN	191.2 ± 17.6^#^	38.7 ± 3.2	69.3 ± 9.5^#^	0.0235 ± 0.0022^#^
TR	211.2 ± 22.3^*^	38.5 ± 5.5	75.2 ± 7.2^*^	0.0257 ± 0.0036^*^

Values were expressed as mean ± SD.

# P < 0.0.05 vs. SN group, and

* *P < 0.05 vs. TN group*.

## Discussion

In the present study, we found increases in the CD40 protein level, NF-κB DNA-binding activity, and concentrations of IL-1β and TNF-α in the jejunal tissues of rats at 24 h after TBI. However, the administration of rosuvastatin partially inhibited CD40 expression, decreased the NF-κB activation, and reduced the concentrations of IL-1β and TNF-α. Moreover, histopathological evaluation confirmed that the TBI-induced damage to the jejunal structures was ameliorated by rosuvastatin.

Previous studies demonstrated that the CD40/CD40L pathway plays a key role in intestinal inflammation by increasing the secretion of multiple pro-inflammatory cytokines and chemokines (20, 21). Although the specific mechanisms underlying how statins inhibit CD40 expression remain poorly understood, several studies have supported the hypothesis that these effects are mediated by nitric oxide synthase (NOS)- or peroxisome proliferator-activated receptor (PPAR)-dependent pathways (11, 22, 23). We hypothesize that the inhibitory effect of rosuvastatin on post-TBI CD40 expression in the jejunum was mediated by the same pathway.

It has been reported that NF-κB, a pivotal cytokine downstream of CD40, plays a fundamental role in regulating cytokine-mediated inflammatory processes (6, 24). The functional importance of NF-κB in acute inflammation is related to its ability to regulate the transcription of numerous genes, such as IL-1β, TNF-α, IL-6, intercellular adhesion molecule (ICAM)-1, and acute phase proteins, which have been shown to be critical in inflammatory processes (25, 26). Increasing evidence has convincingly indicated that corticosteroid hormones, antioxidants, protease inhibitors, and other compounds may treat pathological inflammatory conditions by inhibiting NF-κB activation (27). In our previous research, we demonstrated that progesterone suppressed TBI-induced NF-κB activation in the gut, decreased the intestinal production of pro-inflammatory cytokines, and protected the structures of the ileal mucosa (6). In the present study, we found that rosuvastatin blocked NF-κB activation, and subsequently down-regulated IL-1β and TNF-α levels. The inhibition of NF-κB activation might be attributable to the reduced phosphorylation and degradation of the NF-κB inhibitor protein IκB, as well as the absence of mevalonate caused by inhibiting HMG-CoA reductase (28).

Our study had a notable limitation concerning the focus on rosuvastatin rather than multiple statins. Although researchers have reported that other statins beside rosuvastatin (such as simvastatin and atorvastatin) might exhibit anti-neuroinflammatory effects in animal models of TBI (29, 30), we did not compare these other statins with rosuvastatin. Xu et al. found that acute atorvastatin administration effectively modulated post-TBI neuroinflammation, probably by altering peripheral leukocyte invasion and the alternative polarization of microglia/macrophages (29). Additionally, Chong et al. found that the neuroprotective effect of simvastatin in TBI might be due to its anti-neuroinflammatory effects rather than its cholesterol-lowering effects (30). However, there are few studies on the regulation of post-TBI intestinal inflammation by simvastatin and atorvastatin and, as the aim of our study concerned post-TBI extracranial complications, we did not assess the effects of rosuvastatin on neuroinflammation. We believe that rosuvastatin might share anti-inflammatory effects with the abovementioned statins, as activated neuroinflammation predominantly relies on the activation of peripheral immunocytes, and 70–80% immunocytes are located in the gut associated lymphoid tissue (GALT) (31). However, mechanistic research should now be conducted to determine whether different statins have different effects regarding inflammation.

In conclusion, we found that rosuvastatin has an anti-inflammatory effect, in addition to its ability to reduce cholesterol levels (32). This anti-inflammatory effect might involve both HMG-CoA reductase-dependent and -independent mechanisms (33, 34). Our results suggest that rosuvastatin can partially prevent acute TBI-induced injury to the rat jejunum, probably due to its blockade of the CD40/NF-κB pathway. The specific protection mechanisms require further exploration.

## Data Availability Statement

All datasets generated for this study are included in the article/Supplementary Material.

## Ethics Statement

The animal study was reviewed and approved by the Ethics Committee of Anhui Medical University.

## Author Contributions

All authors contributed extensively to the work presented in this paper. All authors collected data. YH wrote the manuscript draft. XW and LY reviewed and edited the manuscript. CL and WC conducted the experiments. LY and HC revised it critically for important intellectual content.

## Conflict of Interest

The authors declare that the research was conducted in the absence of any commercial or financial relationships that could be construed as a potential conflict of interest.

## Supplementary Material

Supplementary Figure 1 Western blots replicates of CD40 levels in the jejunal tissues for another 5 groups. SN, sham operation + normal saline; TN, TBI + normal saline; TR, TBI + rosuvastatin.
