# Supplementary figures and images for: Rosuvastatin Alleviates Intestinal Injury by Down-Regulating the CD40 Pathway in the Intestines of Rats Following Traumatic Brain Injury
Source: Front Neurol. 2020 Aug 11;11:816. doi: 10.3389/fneur.2020.00816 (PMC7431906; doi:10.3389/fneur.2020.00816)

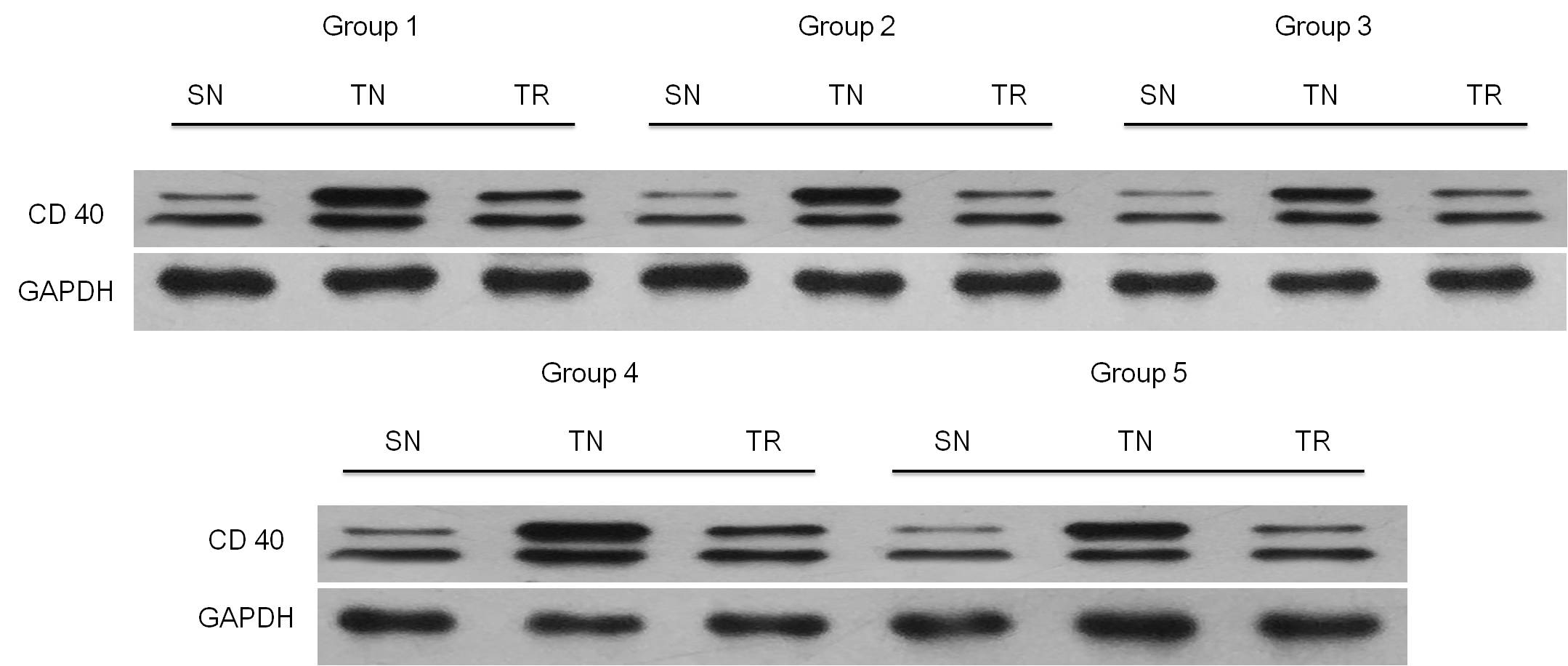

Supplement: Supplementary Figure 1 — Western blots replicates of CD40 levels in the jejunal tissues for another 5 groups. SN, sham operation + normal saline; TN, TBI + normal saline; TR, TBI + rosuvastatin. [file Image_1.JPEG]
